# Supplementary material for: Assessing anxiety, depression and quality of life in patients with peripheral facial palsy: a systematic review
Source: PeerJ. 2020 Dec 1;8:e10449. doi: 10.7717/peerj.10449 (PMC7718791; doi:10.7717/peerj.10449)
Supplement: Supplemental Information 2 [file peerj-08-10449-s002.docx]

**The rationale for conducting the systematic review / meta-analysis:**

The main rationale for conducting this review study was to provide answers regarding the presence of psychological variables in PFP. So far, as we have seen, no study compiles the observational articles regarding the presence of anxiety, depressive symptoms and quality of life in PFP.

**The contribution that it makes to knowledge in light of previously published related reports, including other meta-analyses and systematic reviews:**

It contributes mainly to provide a high level of evidence for the presence of these variables in the PFP. It also highlights the need for further research in this field of patients. Finally, this study adds all published observational articles on this field of study
