# Supplementary material for: Assessing anxiety, depression and quality of life in patients with peripheral facial palsy: a systematic review
Source: PeerJ. 2020 Dec 1;8:e10449. doi: 10.7717/peerj.10449 (PMC7718791; doi:10.7717/peerj.10449)
Supplement: Supplemental Information 3 [file peerj-08-10449-s003.pdf]

Presence of psychological variables in patients with facial paralysis: A systematic review and meta-analysis.

Ferran Cuenca-Martínez, Roy La Touche-Arbizu, Eva Zapardiel-Sánchez, Enrique Carrasco-González

To enable PROSPERO to focus on COVID-19 registrations during the 2020 pandemic, this registration record was automatically published exactly as submitted. The PROSPERO team has not checked eligibility.

Citation

Ferran Cuenca-Martínez, Roy La Touche-Arbizu, Eva Zapardiel-Sánchez, Enrique Carrasco-González. Presence of psychological variables in patients with facial paralysis: A systematic review and meta-analysis.. PROSPERO 2020 CRD42020159843 Available from:
https://www.crd.york.ac.uk/prospero/display_record.php?ID=CRD42020159843

Review question

What psychological variables may be involved in patients with facial paralysis?

Searches

The serche for scientific articles will perform using PubMed, PEDro, Google Scholar and CINAHL.

Types of study to be included

Observational studies and case-control studies.

Condition or domain being studied

Facial paralysis and its psychological variables.

Participants/population

Inclusion criteria: patients with facial paralysis.

Exclusion criteria: patients without facial paralysis.

Intervention(s), exposure(s)

Patients with facial paralysis and with psychological variables associated.

Comparator(s)/control

Healthy subjects.

Main outcome(s)

House-Brackmann Scale, Hospital Anxiety and Depression Scale, Facial Disability Index, Facial Clinimetric Evaluation, Beck Depression Inventory.

* Measures of effect

As given in the source articles.

Additional outcome(s)

Not applicable.

* Measures of effect

Not applicable.

Data extraction (selection and coding)

Observational studies and case-control studies.

Risk of bias (quality) assessment
The risk of bias will be assessed by using the "Modified Newcastle-Ottawa quality assessment scale". This assessment tool consists of a point system for which a study is judged on. It presents 8 items divided

into 3 categories: the selection of the study groups, the comparability of the groups and the ascertainment of either the exposure or outcome of interest for cohort studies. The maximum score a study can receive is 9 points.

Strategy for data synthesis
The search for scientific articles will perform using PubMed (1950 to November 2019), PEDro (1999 to November 2019), CINAHL (1982 to November 2019) and Google Scholar with an end of November, 2019.

Analysis of subgroups or subsets
None.

Contact details for further information
Roy LaTouche-Arbizu
roylatouche@lasallecampus.es

Organisational affiliation of the review
Centro Superior de Estudios Universitarios La Salle, Universidad Autónoma de Madrid, Spain

Review team members and their organisational affiliations
Mr Ferran Cuenca-Martínez. Motion in Brains Research Group, Institute of Neuroscience and Sciences of the Movement (INCIMOV), Centro Superior de Estudios Universitarios La Salle, Universidad Autónoma de Madrid, Spain.
Dr Roy La Touche-Arbizu. Motion in Brains Research Group, Institute of Neuroscience and Sciences of the Movement (INCIMOV), Centro Superior de Estudios Universitarios La Salle, Universidad Autónoma de Madrid, Spain.
Miss Eva Zapardiel-Sánchez. Centro Superior de Estudios Universitarios La Salle, Universidad Autónoma de Madrid, Spain.
Ms Enrique Carrasco-González. Centro Superior de Estudios Universitarios La Salle, Universidad Autónoma de Madrid, Spain.

Type and method of review
Meta-analysis, Systematic review

Anticipated or actual start date
25 November 2019

Anticipated completion date
31 March 2020

Funding sources/sponsors
No funding sources.

Conflicts of interest

Language
English

Country
Spain

Stage of review
Review Ongoing

Subject index terms status
Subject indexing assigned by CRD

Subject index terms
MeSH headings have not been applied to this record

Date of registration in PROSPERO
28 April 2020

Date of first submission
25 November 2019

Stage of review at time of this submission
The review has not started

Stage	Started	Completed
Preliminary searches	No	No
Piloting of the study selection process	No	No
Formal screening of search results against eligibility criteria	No	No
Data extraction	No	No
Risk of bias (quality) assessment	No	No
Data analysis	No	No

The record owner confirms that the information they have supplied for this submission is accurate and complete and they understand that deliberate provision of inaccurate information or omission of data may be construed as scientific misconduct.

The record owner confirms that they will update the status of the review when it is completed and will add publication details in due course.

Versions
28 April 2020

PROSPERO
This information has been provided by the named contact for this review. CRD has accepted this information in good faith and registered the review in PROSPERO. The registrant confirms that the information supplied for this submission is accurate and complete. CRD bears no responsibility or liability for the content of this registration record, any associated files or external websites.
